# Using Item Response Theory to evaluate the psychometric characteristics of questions in a Brazilian programme and the performance of dental teams in primary care

**DOI:** 10.1371/journal.pone.0217249

**Published:** 2019-05-31

**Authors:** Suellen R. Mendes, Renata C. Martins, Juliana V. M. Mambrini, Antônio Thomaz G. Matta-Machado, Grazielle C. M. Mattos, Jennifer E. Gallagher, Mauro H. N. G. Abreu

**Affiliations:** 1 Graduate Program in Dentistry, Faculty of Dentistry, Universidade Federal de Minas Gerais, Belo Horizonte, Minas Gerais, Brazil; 2 Department of Community and Preventive Dentistry, Faculty of Dentistry, Universidade Federal de Minas Gerais, Belo Horizonte, Minas Gerais, Brazil; 3 René Rachou Institute, FIOCRUZ, Belo Horizonte, Minas Gerais, Brazil; 4 Department of Preventive and Social Medicine, Faculty of Medicine, Universidade Federal de Minas Gerais, Belo Horizonte, Minas Gerais, Brazil; 5 Kings College London, Faculty of Dentistry, Oral & Craniofacial Sciences, Centre for Host Microbiome Interactions, London, United Kingdom; University of Bern, SWITZERLAND

## Abstract

**Objectives:**

First, to assess the psychometric properties of key questions included in a public sector evaluation of primary dental care in Brazil; and second, to evaluate the performance of dental teams in relation to these items.

**Methods:**

Secondary analysis of a national primary care dataset monitoring quality and access to dental care. Data were collected through face-to-face interviews with representatives of dental teams participating in the ‘National Programme for Improving Access and Quality of Primary Care’. Twenty-three mandatory questions about the dentists’ reported delivery of dental procedures were included in the analysis. Item Response Theory (IRT) modelling was applied to measure the psychometric properties of the instrument—level of difficulty and discrimination parameter of each item—and then to estimate dental team performance scores based on these parameters. Based on IRT, possible scores ranged from -4 to +4.

**Results:**

Three of the 23 mandatory items were removed due to poor internal consistency, resulting in a scale of 20 items for assessing dental team performance. The results showed variation in procedures delivered by the dental teams; whilst more than a half of the procedures were executed by at least 80% of the dental teams, those relating to dentures (partial/total) and frenectomy (lingual/labial) were performed by less than 30%. Amongst the 20 items included in the model, those related to partial/total dentures and oral cancer follow-up presented higher levels of difficulty and were less frequently provided. The items relating to the treatment of deciduous teeth and access to the dental pulp of permanent teeth had the highest discrimination parameters and, consequently, greater weight in the performance’s score estimation; therefore, dental teams that did not perform these items had the lowest performance scores. In the present study, dental team performance scores ranged from -3.66 to +1.87 with a mean/median of -0.06/+0.01.

**Conclusion:**

The findings suggest that whilst the items within the instrument demonstrated some potential to discriminate between poor and very poor teams, they were ineffective in discriminating between poor and good teams. Whilst Brazilian dental teams perform many mandatory procedures, variation in the nature of their delivery of care requires further investigation to enhance service provision to the population.

## Introduction

Brazil is a large country that covers approximately 47% of South American landmass, with a population estimate of 209 million inhabitants across five geographical regions (north, northeast, centre-west, southeast and south) and marked socioeconomic disparities [1]. Since 1988, the Brazilian population has been provided with access to all levels of health care through the Brazilian National Health System (known as Sistema Único de Saúde and abbreviated to ‘SUS’), which is free at the point of delivery. It is therefore one of the biggest health care systems in the world, serving approximately 60% of the Brazilian population; none-the-less, whilst it is open to all, however, some people choose to seek private care [2,3].

Inclusion of dentistry in SUS only occurred in the year 2000 and this was followed by the creation of a National Oral Health Policy (NOHP) in 2003. A great increase in the number of dental facilities and enhanced qualification of the dental workforce was observed thereafter. SUS primary health care (PHC) dentistry, which is similar to routine care provided through the National Health Service (NHS) in England, includes preventive, restorative/prosthetic, endodontics and surgical procedures, with referral to specialists for more complex conditions such as impacted third molar extraction and serious conductions such as suspected oral cancer [4,5]. There are about 309,100 active dentists in Brazil, almost half of whom work in the public sector across primary, secondary and tertiary care [6,7]. Currently, there are 24,057 dental health teams (7.8% of the all active Brazilian dentists) working at PHC units delivering primary care in the public sector [8].

Oral health in Brazil appears to be improving. The most recent Brazilian oral health survey reported a decrease in dental caries amongst 12 years-old children and adolescents (aged 15–19 years) and increase uptake of dental care by adults. However, the same survey revealed that dental caries prevalence in the primary dentition remains high and suggested that 68.8% of adults and 92.7% of elderly people require rehabilitation which include total/partial dentures and crowns/bridges. Although construction of dentures and crown and bridgework are not mandatory in the Brazilian PHC, the delivery of dentures in particular is strongly recommended by the MofH, as there are about 17 million edentulous people nationally. Furthermore, there is a huge inequality within, and between, Brazilian geographical regions [3,9].

With the aim of monitoring health service to improve access and quality, the Brazilian Ministry of Health (MofH) launched the ‘National Programme for Improving Access to and Quality of Primary Care’ (PMAQ-AB). It involves a cross-sectional public sector survey, launched in 2011, through a partnership between the Brazilian MofH and six Brazilian education and research institutions. This voluntary programme, based on a ‘Pay for Performance’ system, may be followed by all PHC teams of SUS, including the dental teams. Teams participating in the programme, and demonstrating good outcomes, receive a financial incentive and can proceed to a new evaluation cycle. This national service evaluation has therefore generated a large dataset with information on the majority of Brazilian PHC teams [10–12], which is available for analysis.

To date, the PMAQ-AB has conducted two evaluation cycles, first in 2011/2012 and second in 2013/2014. Evaluating data from the first cycle, Reis et al. (2015; 2017), reported that a large variety of dental procedures were delivered by dental teams participating in the evaluation (n = 12,404), but items such as dental prostheses were not [13,14].

Given the importance of continuous assessment in health to the organisation and supply of services [15,16], data from the first and second evaluation cycles of PMAQ-AB have been analysed [13,14,17]. Provisional analysis reporting dentists’ productivity across 23 standard primary dental care procedures suggested there is variation in dental team performance [17]. Now that this form of monitoring has been accepted, further research is required to evaluate the psychometric properties of the PMAQ-AB instrument, and its ability to discriminate between teams.

Therefore, the present study aimed first to assess the psychometric properties of key questions included in the public sector evaluation on primary dental care in Brazil; and second, to evaluate the reported performance of the dental teams in relation to these items.

## Methodology

### Study Design

This study was submitted to, and approved by, the National Ethics Research Council and by the Research Ethics Committee of the Federal University of Minas Gerais (Protocol No. 02396512.8.0000.5149; Approval No. 2004382) in order to gain access to the Brazilian MofH database.

The present study involved secondary analysis of data from the PMAQ-AB dataset (2nd cycle), conducted between 2013 and 2014. Participation was open to all 23,150 dental teams working at PHC at that time. Out of a total of 19,946 applicants, 1,832 were excluded by the PMAQ-AB evaluation criteria, as they did not have fully functioning dental team and equipment, resulting in the data from 18,114 dental teams being evaluated in the second cycle of the programme [8], and available for analysis in this study.

### Data Collection

The PMAQ-AB programme comprises four phases—agreement, development, external evaluation, and re-contractualisation—which complement each other and form a continuous evaluation cycle of PHC teams [11]. The external evaluation phase is comprised of a questionnaire applied at the PHC units, together with verification. The questionnaires were constructed on the principles of PHC and Donabedian’s model for health services evaluation, examining structure, process and outcomes [18]; and include questions concerning the dental facilities structure, dental instruments, dental procedures executed, dentists’ profile, management and service organization. The questions primarily involved dichotomous responses (yes/no) and were answered in a face-to-face interview with a representative dentist from each PHC unit [11,12].

The service evaluation involved a team of 989 interviewers, all senior health professionals, who underwent a 40-hour training programme regarding PHC in SUS, survey methods, and PMAQ-AB questionnaires to enable then to conduct this survey nationwide. All interviewers underwent formal evaluation to assess their abilities prior to commencing the study. The Brazilian MofH developed a mobile app with the relevant questions which sent responses to a central online database. Initially the MofH made these data available to the partner institutions; and, thereafter, publicly available online [11,12]. The PMAQ-AB questionnaire was constructed specifically to evaluate the Brazilian National Health System and face validity of the instrument was determined by experts in the field; with certain responses requiring supporting evidence for the purpose of validation, such as clinical records of suspected/confirmed oral cancer cases.

**Fig 1:**
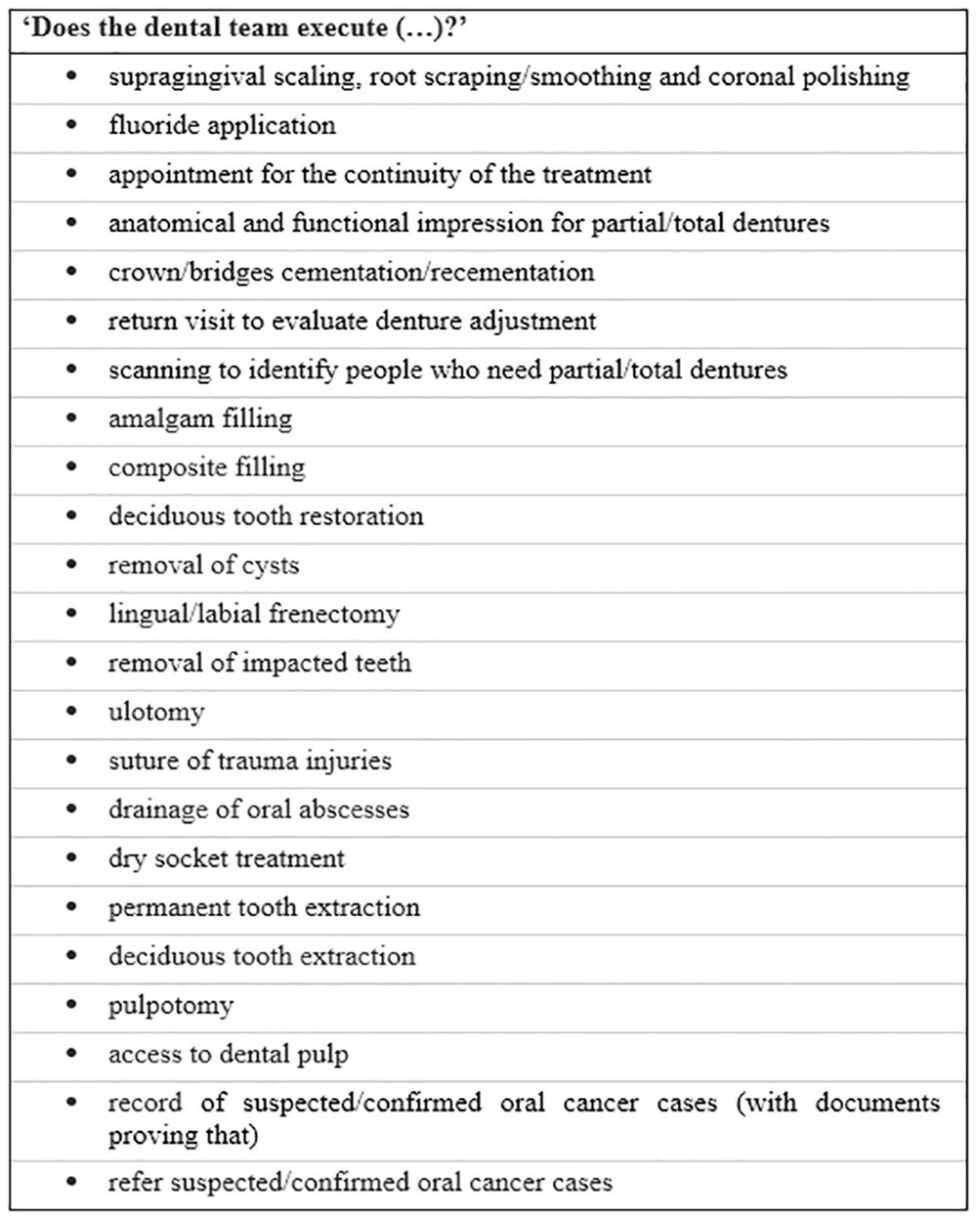
Questions related to the dental procedure’s execution asked to the PHC dentist in a face-to-face interview.

In the present study, we assessed questions related to the delivery of 23 mandatory dental procedures in the PHC unit (Fig 1). All these questions were part of the external evaluation phase of PMAQ-AB, Module VI, involving data gained in face-to-face interview with the dentist at the PHC unit. Whilst the overall Module VI of PMAQ-AB had 108 questions, the 23 mandatory items were selected to be part of the study since they are primary health care procedures routinely executed and determined as essential in Brazil.

### Statistical analysis

The psychometric characteristics of the instrument and dental team performance related to primary care services in dentistry were analyzed using Item Response Theory (IRT). IRT involves a set of mathematical models that relate the probability of an individual’s response to an item and its latent trait (or unknown construct). The latent construct is a characteristic that cannot be measured directly, as attitude, satisfaction, and proficiency [19–21]. The unobservable variable, i.e. dental team performance, was estimated based on the dentists’ report about the delivery of each dental procedure.

IRT modelling makes possible more detailed analysis of the items used to construct the measurement: level of difficulty and discrimination ability. Thus, when modelling, it is not the number of dental procedures performed by the dental teams that determine their performance, but the weighting for the level of difficulty (estimated based on the frequency of delivery) and the ability to discriminate between items. The scores attributed to each dental team theoretically can range from -4 to +4 [19–22].

Amongst existing IRT models, the graded response model proposed by Samejima (1969) was chosen. This model is usually applied in questionnaires of ordered responses, which reflect personal positioning, so there are no right or wrong answers, and it can be applied to dichotomous or polytomic variables [23].

Descriptive analyses of dental procedures were first undertaken to provide an overview of the data. Thereafter, to confirm the feasibility of applying the IRT, the first eigenvalue was calculated from the decomposition of the polychoric correlation matrix, verifying its domain. In addition, internal consistency of the scale was assessed using Cronbach’s alpha [19,21,24].

The discrimination parameter (a); and, level of difficulty (b), of each procedure evaluated were calculated. Based on the findings, each dental team received a score based on the IRT modeling. For each dental procedure included in this performance estimation, the frequency of execution was calculated, total and according to groups of dental teams with ‘high’, ‘medium’ and ‘low’ scores. Besides that, a test information curve aggregating all items was made to identify the score interval about which the present instrument provides information [22].

Data were entered and organised in Stata Software (StataCorp. 2015. Stata Statistical Software: Release 14. College Station, TX: StataCorp LP). R Software was used to analyse the data (descriptive analysis and correlation matrix to check the IRT assumptions); and latent trait model (LTM) package was used for the IRT model adjustment [25].

## Results

The correlation of the 23 items from the original scale presented with a low Cronbach’s alpha coefficient, and three items were therefore removed. The total Cronbach’s alpha of the 20 remaining items was 0.66, which is considered acceptable internal consistency [24]. It is important to note that the dental procedures which were unreliable in the model were also less commonly provided: ‘anatomical and functional impression for dentures/prostheses’ (8.2%), ‘removal of cysts’ (21.4%), and ‘removal of impacted teeth’ (30.1%).

The correlation matrix for the 20 items ranged from 0.02 (‘frenectomy’ and ‘reference to forward cases of oral cancer’ items) to 0.92 (‘deciduous tooth extraction’ and ‘permanent tooth extraction’ items). The largest eigen value which indicates 36.8% of the total variance was substantially higher than the second largest (9.9%) indicating the uni-dimensionality of the instrument.

The findings suggest that more than half of the procedures (70%) were executed by 80% or more of the dental teams. Descriptive analysis showed the biggest floor effect for the item ‘return visit to evaluate denture adjustment’ (it was not performed by 87.7% of the dental teams), whilst the biggest ceiling effect was for the item ‘fluoride application’ (it was performed by 98.6% of the dental teams).

Table 1 shows the 20 dental PHC procedures included in the evaluation of dental team performance through the IRT model. For each one, the frequency of accomplishment, the discrimination parameter (a), and level of difficulty (b), are presented. Dental procedures are arranged in descending order regarding the total frequency of accomplishment, according to each dental procedure’s category: preventive procedures, restorative/prosthetic procedures, surgical procedures, endodontic procedures, and cancer monitoring.

**Table 1:** Performance of each item provided in primary dental care: Total and categorized in groups of dental teams with the lowest, medium and highest scores; group’s frequency range; (a) discrimination parameter; and (b) reported difficulty level, Brazil, 2013–2014.

Items	Frequency of accomplishment^1^^,^^2^					Discrimination ability (a)	Level of difficulty (b)
	Total (%)	Lowest scores (%)	Medium scores (%)	Highest scores (%)	Groups’ range (%)		
**Preventive Procedures**							
Fluoride application	98.6	96.7	99.4	99.9	3.2	1.475	-3.615
Supragingival scaling, root scraping/smoothing and coronal polishing	96.9	91.5	99.3	99.9	8.4	1.779	-2.668
Appointment for the continuity of treatment	91.9	84.6	93.1	98.0	13.4	0.706	-3.716
**Restorative/Prosthetic Procedures**							
Deciduous tooth restoration	98.6	95.8	99.9	100.0	4.2	2.652	-2.679
Composite filling	97.8	94.3	99.4	99.8	5.5	1.611	-3.083
Amalgam filling	89.3	79.6	91.9	96.4	16.8	0.768	-3.050
Scanning to identify people who need partial/total dentures	52.5	36.8	51.0	69.7	32.9	0.605	-0.177
Crown/bridges cementation/recementation	28.2	8.4	18.3	57.9	49.5	1.029	1.097
Return visit to evaluate denture adjustment	12.3	4.5	6.6	25.9	21.4	0.855	2.594
**Surgical Procedures**							
Deciduous tooth extraction	98.5	95.7	99.8	100.0	4.3	2.268	-2.827
Permanent tooth extraction	98.0	94.6	99.4	100.0	5.4	1.846	-2.910
Dry socket treatment	90.2	73.0	97.7	99.9	26.9	1.742	-1.817
Drainage of oral abscesses	87.8	69.4	94.7	99.5	30.1	1.332	-1.902
Suture of trauma injuries	80.0	61.4	82.9	95.6	34.2	0.848	-1.858
Ulotomy	65.8	33.8	68.9	94.7	60.9	1.237	-0.683
Lingual/labial frenectomy	27.2	11.9	19.5	50.3	38.4	0.724	1.509
**Endodontic procedures**							
Access to dental pulp	88.1	66.9	97.7	99.8	32.9	1.925	-1.579
Pulpotomy	83.3	56.6	93.9	99.5	42.9	1.777	-1.338
**Cancer Monitoring**							
Refer suspected/confirmed oral cancer cases	80.0	63.2	82.6	94.4	31.2	0.756	-2.044
Record of suspected/confirmed oral cancer cases (with documents proving that)	22.8	8.1	15.8	44.5	36.4	0.782	1.749

^1^ Dental procedures delivered at least once by the dental team;

^2^Lowest scores from -3.66 to -0.34; medium scores from -0.35 to 0.29; highest scores from 0.30 to 1.87.

Regarding the discrimination parameter (a), the dental procedures with the biggest scores and thus, with greater weight in the dental team performance estimation were ‘deciduous tooth filling’ (2.652), ‘deciduous tooth extraction’ (2.268), ‘access to dental pulp’ (1.925), ‘permanent tooth extraction’ (1.846), and ‘supragingival scaling, root planning/debriment and coronal polishing’ (1.779). Concerning the difficulty parameter (b), the dental procedures that showed the highest values (and less frequently performed) were ‘return visit to evaluate denture adjustment’ (2.594), ‘record of suspected/confirmed cases of oral cancer’ (1.794), ‘lingual/labial frenectomy’ (1.509), and ‘crown/bridge cementation/re-cementation’ (1.077).

Although the discriminatory potential is not necessarily related to the frequency of provision of the dental procedure, it is interesting to observe that the more discriminating procedures had high frequencies of delivery (>80%). It suggests that whether teams perform these procedures, or not, is an efficient way to discriminate between teams with low scores (which deliver these procedures somewhat more frequently than the very low scores) from teams with very low scores (which do not deliver them).

Table 2 shows the frequency of execution of the evaluated dental procedures according to each Brazilian Geographical Region and, altogether the dental procedures were more frequently executed in the South and Southeast regions whilst were less commonly performed in the North and Northeast regions.

**Table 2:** Performance of each item provided in primary dental care according to the Brazilian Geographical Regions—North, Northeast, Centre-West, Southeast and South. Brazil, 2013–2014.

Items	North (n = 1,263) Yes (%)	Northeast (n = 7,700) Yes (%)	Centre-West (n = 1,572) Yes (%)	Southeast (n = 5,027) Yes (%)	South (n = 2,552) Yes (%)
**Preventive Procedures**					
Fluoride application	1,228 (97.2)	7,601 (98.7)	1,547 (98.4)	4,960 (98.7)	2,530 (99.1)
Supragingival scaling, root scraping/smoothing and coronal polishing	1,160 (91.8)	7,486 (97.2)	1,453 (92.4)	4,949 (98.4)	2,503 (98.1)
Appointment for the continuity of treatment	1,091 (86.4)	6,805 (88.4)	1,490 (94.8)	4,909 (97.7)	2,344 (91.8)
**Restorative/Prosthetic Procedures**					
Deciduous tooth restoration	1,213 (96.0)	7,564 (98.2)	1,550 (98.6)	4,984 (99.1)	2,540 (99.5)
Composite filling	1,214 (96.1)	7,488 (97.2)	1,545 (98.3)	4,948 (98.4)	2,523 (98.9)
Amalgam filling	747 (59.1)	7,115 (92.4)	1,380 (87.8)	4,698 (93.5)	2,240 (87.8)
Scanning to identify people who need partial/total dentures	470 (37.2)	4,102 (53.3)	704 (44.8)	2,935 (58.4)	1,292 (50.6)
Crown/bridges cementation/recementation	146 (11.6)	1,434 (18.6)	343 (21.8)	2,250 (44.8)	936 (36.7)
Return visit to evaluate denture adjustment	48 (3.8)	813 (10.6)	137 (8.7)	851 (16.9)	382 (15.0)
**Surgical Procedures**					
Deciduous tooth extraction	1,228 (97.2)	7,574 (98.4)	1,539 (97.9)	4,972 (98.9)	2,529 (99.1)
Permanent tooth extraction	1,227 (97.1)	7,559 (98.2)	1,505 (95.7)	4,930 (98.1)	2,525 (98.9)
Dry socket treatment	1,091 (86.4)	6,712 (87.2)	1,346 (85.6)	4,748 (94.4)	2,435 (95.4)
Drainage of oral abscesses	1,117 (88.4)	6,234 (81.0)	1,411 (89.8)	4,716 (93.8)	2,430 (95.2)
Suture of trauma injuries	1,023 (81.0)	5,824 (75.6)	1,309 (83.3)	4,066 (80.9)	2,263 (88.7)
Ulotomy	698 (55.3)	4,642 (60.3)	970 (61.7)	3,675 (73.1)	1,930 (75.6)
Lingual/labial frenectomy	336 (26.6)	1,921 (24.9)	424 (27.0)	1,413 (28.1)	835 (32.7)
**Endodontic procedures**					
Access to dental pulp	964 (76.3)	6,326 (82.2)	1,415 (90.0)	4,797 (95.4)	2,457 (96.3)
Pulpotomy	873 (69.1)	5,858 (76.1)	1,340 (85.2)	4,705 (93.6)	2,317 (90.8)
**Cancer Monitoring**					
Refer suspected/confirmed oral cancer cases	841 (66.6)	5,924 (76.9)	1,157 (73.6)	4,332 (86.2)	2,243 (87.9)
Record of suspected/confirmed oral cancer cases (with documents proving that)	99 (7.8)	1,250 (16.2)	245 (15.6)	1,702 (33.9)	832 (32.6)

The scores representing the performance of each dental team (n = 18,114) ranged from -3.66 to +1.87. The overall mean was -0.06 (SD = 0.82), and median was 0.01, showing left (negative) skewed. Although no cut off values were determined to these scores, in the present study they were divided by a mathematical appraisal, thus classifying the teams evaluated in ‘low scores’ (ranging from -3.66 to -0.34), ‘medium’ (ranging from -0.35 to +0.29) and ‘high’ (ranging from +0.30 to +1.87). They were composed by 6,044, 6,033 and 6,037 dental teams, respectively and, differences between teams with low, medium and high scores and procedures with the greatest range between groups, were those with the lowest total frequencies.

The findings presented in Table 3 show that more than a half of dental teams from the North region and almost half of dental teams from Northeast had lowest performance scores, whilst half of dental teams from South and Southeast regions had the highest performance.

**Fig 2:**
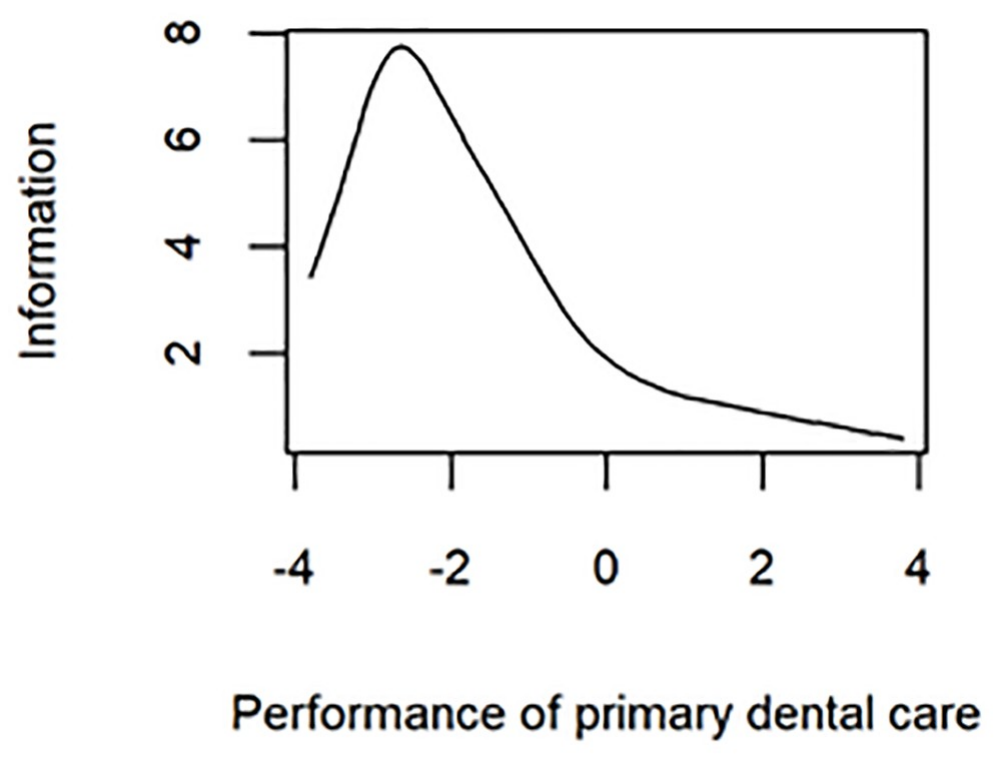
Test information curve, showing the score interval about which the present instrument provides more information.

The test information curve, presented in Fig 2, indicates that dental procedures included in the PMAQ-AB instrument and analysed in the present study provide more information about the dental teams in the negative end of scores spectrum, especially those between -2 and -3.

**Table 3:** Frequency of dental teams with low, medium and high scores according to each Brazilian Geographical Region—North, Northeast, Centre-West, Southeast and South. Brazil, 2013–2014.

	Lowest Score Teams N (%)	Medium Score Teams N (%)	Highest Score Teams N (%)	Total
**North**	712 (56.4)	398 (31.5)	151 (12.0)	**1,263**
**Northeast**	3,383 (43.9)	2,576 (33.5)	1,741 (22.6)	**7,700**
**Centre-West**	589 (37.5)	536 (34.1)	447 (28.4)	**1,572**
**Southeast**	900 (17.9)	1,665 (33.1)	2,462 (49.0)	**5,027**
**South**	460 (18.0)	858 (33.6)	1,234 (48.4)	**2,552**
**Total**	**6,044**	**6,033**	**6,037**	**18,114**

## Discussion

The items included in the estimation of dental team performance are mandatory in the Brazilian PHC dentistry, although the dental teams are not forced to perform items which are beyond their competence. Whilst most of the dental procedures were reportedly executed by most dental teams; it has been possible to observe through the two PMAQ-AB cycles (2011/2012 and 2013/2014) that the additional procedures included after NOHP was introduced had the lowest individual performance frequencies, and so present higher levels of difficulty. Most notably they related to the provision of partial/total dentures and oral cancer monitoring. Furthermore, it is of great concern that certain dental teams did not even meet the requirements to participate in the evaluation and they clearly need urgent investigation and support to ensure that they are fit for purpose of PHC.

In relation to the data which represents the majority of PHC nationally, it has been suggested that inadequate provision of dentures may be related to the unequal distribution of SUS regional prosthodontic laboratories throughout the country; even with the care almost restricted to partial/total dentures [26]. It is of concern that dental rehabilitation may not be adequately provided by the public sector, as the need for total/partial dentures and crown/bridges is still great among adults and the elderly [27], and our analysis suggests that these items are not available across SUS dental teams and supports the findings of Cunha et al. [26]. It suggests that the population may be forced to use the private sector, or remain on waiting lists for long time, in order to receive necessary dental prosthetic treatment, which increases inequalities. This is an area for urgent identification of the barriers to dental care for adults, together with the educational and training needs of the dental workforce so that service barriers can be overcome to ensure that the population has access to prosthodontics [26].

Considering that the oral cancer corresponds to 1–2% of the diagnosed cancers and only 50% of these cases are identified in the initial stages [28,29], it is of concern that less than 23% of the dental teams reported having records of suspected and confirmed oral cancer cases. Although 80% of them reported having a specialized center to refer these patients, not having registers can indicate a weak bond between the primary and secondary care or a failure in the identification of initial cancer lesions. On the other hand, it can represent the low frequency of patients that are diagnosed with suspected/confirmed oral lesions in the working life of dentist [29]. It is important to ensure that primary care dentists have the appropriate education and training in relation to the identification and referral of patients with per-malignant and malignant lesions in a timely manner, which can increase the success of the treatment.

Although some dental procedures were included at the PHC dentistry of SUS after the NOHP, what is observed in practice is that the traditional dental care model, based on direct restorations and extractions, is still being prioritised. Whilst there is undoubtably significant unmet need and requirement for routine procedures, it is important to provide a comprehensive dental treatment with more complex procedures. Also, this traditional model may reflect dental education and training which is still focused merely on resolution of the current dental problems of the majority of the population [26,29], rather than also extending to preventive care.

The dental care consequences of socioeconomic disparities throughout the country were evidenced by the differences in the delivery of dental procedures and in the performance scores of the dental teams between the Brazilian Geographical Regions, which suggest that additional resources should be allocated to the regions that presented with low performance scores, especially North and Northeast regions [13,26]. However, it is important to highlight that the access to health care is determined not only by the service availability and professional judgment about patient need of care, but also refers to the initiation by the patient into the process of utilising the service. “Gaining access” is also influenced by patient’s perceived need for care, financial barriers and previous experience in the health service [30–32], which is influenced by social status [32].

It is interesting to note that the dental procedures with the highest discrimination parameters were frequently performed by dental teams; and, consequently, presented low levels of difficulty, especially those related to children’s care. Since most dental teams reported delivering mandatory procedures considered to be in high need, the fact that some teams did not provide these procedures is vitally important in differentiating teams that performed the bare minimum of care from those well below expectations. In fact, if some dental teams do not provide those commonplace procedures, we advocate that it is necessary to examine the reasons behind this. It may be due to lack of skills amongst the dentists or inadequate equipment and facilities at the dental surgery, which suggests that this should be addressed as a matter of urgency. In both cases, public policies are necessary to expand access to dental care for the Brazilian population, ensuring the equity and the quality of the offered care [33].

Given our findings, it is necessary to emphasise the importance of ensuring that the dental workforce matches Brazilian population oral health needs as closely as possible. Either through facilities development, expansion of the (number of) dental teams working in the PHC, distribution through the country, or, possibly further incentives to encourage them to take the necessary training to enable them to perform more needed dental procedures. This action could not only help to address the missing procedures amongst dental primary care, but also, and very importantly, results in improved population oral health [34,35].

The dental procedures included in the evaluation of dental team performance were not good in discriminating between dental teams with high and low scores. However, the dental procedures evaluated are those necessary to meet the needs and demands of the Brazilian population. In the view of the authors, all 20 dental procedures evaluated should be maintained at PHC, besides insurance of dental care access to all the Brazilian population. It is important to highlight that the PMAQ-AB instrument covers other aspects of PHC services besides dental procedures executed, but an improvement of the instrument may be necessary to future evaluation cycles.

A further avenue would be to evaluate patient oral health outcomes (using the FDI–ICHOM Standard Set of Adult Oral Health Measures, for example) [36] and also identify if they are receiving the proper dental treatment to meet their needs. Furthermore, questions should be included, to examine the why problems exist and how they can be addressed including team knowledge and skills, facilities/equipment and low demand by the local population and how secondary care can assist.

The present study was based on a secondary dataset of the second evaluation cycle of PMAQ-AB; and, municipal managers selected participating dental teams—probably those with the best structures and organisation—which can represent not only a limitation of the study, but a potential increase in the primary health care inequality. It may, therefore, be that the weakest teams did not or could not participate in the survey. Despite this fact, this work represents an important assessment of Brazilian PHC dentistry nationally. More studies on the dental teams that were not qualified for PMAQ evaluation should be also conducted.

Improvements in the PMAQ-AB instrument should be done for future evaluation cycles. Complementary open questions would be important to find out why some dental procedures were not delivered.

## Conclusion

The findings suggest that items within the SUS evaluation instrument demonstrated some potential to discriminate between poor teams from the very poor ones and ineffective in discriminating the teams considered poor from the good ones. Whilst Brazilian dental teams perform many mandatory procedures, variation in the delivery of care requires further investigation to enhance service provision to the population.

## Supporting information

S1 Dataset (SAV)
